# A novel dynamic network imaging analysis method reveals aging-related fragmentation of cortical networks in mouse

**DOI:** 10.1162/netn_a_00191

**Published:** 2021-06-21

**Authors:** Daniel A. Llano, Chihua Ma, Umberto Di Fabrizio, Aynaz Taheri, Kevin A. Stebbings, Georgiy Yudintsev, Gang Xiao, Robert V. Kenyon, Tanya Y. Berger-Wolf

**Affiliations:** Department of Molecular and Integrative Physiology, University of Illinois at Urbana-Champaign, Champaign, IL, USA; Neuroscience Program, University of Illinois at Urbana-Champaign, Champaign, IL, USA; Beckman Institute for Advanced Science and Technology, Urbana, IL, USA; Department of Computer Science, University of Illinois at Chicago, Chicago, IL, USA; Department of Computer Science, University of Illinois at Chicago, Chicago, IL, USA; Department of Computer Science, University of Illinois at Chicago, Chicago, IL, USA; Neuroscience Program, University of Illinois at Urbana-Champaign, Champaign, IL, USA; Beckman Institute for Advanced Science and Technology, Urbana, IL, USA; Neuroscience Program, University of Illinois at Urbana-Champaign, Champaign, IL, USA; Beckman Institute for Advanced Science and Technology, Urbana, IL, USA; Department of Molecular and Integrative Physiology, University of Illinois at Urbana-Champaign, Champaign, IL, USA; Beckman Institute for Advanced Science and Technology, Urbana, IL, USA; Department of Computer Science, University of Illinois at Chicago, Chicago, IL, USA; Department of Computer Science, University of Illinois at Chicago, Chicago, IL, USA; Current affiliation: Department of Computer Science and Engineering, The Ohio State University, Columbus, OH, USA

**Keywords:** Network analysis, Aging, Auditory cortex, Motor cortex, NMDA, Random forest, Presbycusis

## Abstract

Network analysis of large-scale neuroimaging data is a particularly challenging computational problem. Here, we adapt a novel analytical tool, the community dynamic inference method (CommDy), for brain imaging data from young and aged mice. CommDy, which was inspired by social network theory, has been successfully used in other domains in biology; this report represents its first use in neuroscience. We used CommDy to investigate aging-related changes in network metrics in the auditory and motor cortices by using flavoprotein autofluorescence imaging in brain slices and in vivo. We observed that auditory cortical networks in slices taken from aged brains were highly fragmented compared to networks observed in young animals. CommDy network metrics were then used to build a random-forests classifier based on NMDA receptor blockade data, which successfully reproduced the aging findings, suggesting that the excitatory cortical connections may be altered during aging. A similar aging-related decline in network connectivity was also observed in spontaneous activity in the awake motor cortex, suggesting that the findings in the auditory cortex reflect general mechanisms during aging. These data suggest that CommDy provides a new dynamic network analytical tool to study the brain and that aging is associated with fragmentation of intracortical networks.

## Author Summary

In this work, we introduce a novel technique (the community dynamic interference method, CommDy) which is based on social network theory, to analyze neural data. We use CommDy to analyze imaging data from the cerebral cortex of awake mice and mouse brain slices. In each case, data were obtained from young and aged mice. Using CommDy, we found that in both types of data, aging is associated with a fragmentation of cortical circuitry. Specifically, the strength of intracortical connectivity diminishes with aging, leaving fragmented cortical networks. Thus, CommDy is a novel tool now available to the neuroscience community, and has revealed aging-related pathology of cortical networks.

## INTRODUCTION

Normal aging is associated with a gradual loss of cognitive function (Glisky, 2007; Horn, 1982; Horn & Cattell, 1967; Salthouse, 2003; Todd, Andrews, & Conlon, 2019). The mechanisms responsible for this cognitive loss are not yet known, but given the aging of the population (Beard, Officer, & Cassels, 2016), it will be important to understand how brain networks fail with aging. Structural changes in the aging brain have been investigated and are characterized by changes in cortical thickness (Salat et al., 2004; Thambisetty et al., 2010), synaptic density (Huttenlocher, 1979; Liu, Erikson, & Brun, 1996; Masliah, Mallory, Hansen, DeTeresa, & Terry, 1993), and selective loss of inhibitory interneurons (Caspary, Ling, Turner, & Hughes, 2008; Rozycka & Liguz-Lecznar, 2017). Less well characterized are functional changes in cortical physiology with aging, such as changes in functional connectivity. Functional connectivity between brain regions can change rapidly over time (Hutchison et al., 2013; Robinson, Atlas, & Wager, 2015), is not easily predictable from anatomical connectivity (Vanduffel, Payne, Lomber, & Orban, 1997), and is altered in several different pathological states (Damaraju et al., 2014; Damoiseaux, Prater, Miller, & Greicius, 2012; Schmidt, Carpenter-Thompson, & Husain, 2017; Supekar, Menon, Rubin, Musen, & Greicius, 2008). In addition, cortical networks appear particularly vulnerable to aging and demonstrate diminished network-level functional connectivity over the lifespan (Andrews-Hanna et al., 2007; Ash et al., 2016; Battaglia et al., 2020; Tomasi & Volkow, 2012; Wen et al., 2020). Furthermore, aging-related disruptions in functional connectivity correlate with declines in cognitive performance (Onoda, Ishihara, & Yamaguchi, 2012). However, current approaches to examine functional connectivity typically average data over long periods of time relative to timescales relevant for cognitive functions and do not provide a clear mechanism to characterize the dynamics of functional connectivity over time. Therefore, techniques that are able to extract the dynamics of functional connectivity from brain imaging data will be of great value to those studying the impact of aging on the brain and to the broader neuroscience community.

Network analysis tools are emerging approaches to understand functional connectivity of the brain (Ash et al., 2016; Bullmore & Sporns, 2009; Chen, Cai, Ryali, Supekar, & Menon, 2016; Karwowski, Vasheghani Farahani, & Lighthall, 2019; Rossini et al., 2019; Sporns, 2013). Unfortunately, network analysis of brain imaging data has proven to be a particularly challenging computational problem. Brain activity is intrinsically highly dynamic, whereby functional associations between neurons and brain regions ebb and flow as the organism’s level of arousal or focus of attention changes. Many previous network methodologies, particularly those based on blood flow signals, relied on collecting time series over long periods of time to build maps (Beckmann, DeLuca, Devlin, & Smith, 2005; V. Calhoun et al., 2001; Vincent et al., 2007), and the dynamic information extracted from these networks is lost. There have been recent attempts to ameliorate this problem by examining functional connectivity over time. These approaches often, though not always (W. H. Thompson, Richter, Plavén-Sigray, & Fransson, 2018), consisted of choosing a window of time for analysis and sliding this window along the period of data acquisition (V. D. Calhoun, Miller, Pearlson, & Adalı, 2014; Pedreschi et al., 2020; G. J. Thompson et al., 2013). Some of the problems with these approaches have been (a) the analysis windows (typically about 30–60 s) are still long relative to the cognitive processes of interest that evolve over hundreds of milliseconds; (b) choosing smaller windows tends to diminish signal-to-noise (SNR) ratios or are employed on datasets with lower spatial resolution, such as magnetoencephalography (Brookes et al., 2016); and (c) noise may be nonstationary and therefore introduce spurious “networks” along the time course of analysis (Hutchison et al., 2013).

Previous investigators have suggested that complex dynamic networks, be they populations of neurons, large ecosystems, or social networks, share common underlying organizational motifs (Bullmore & Sporns, 2009; Holme & Saramäki, 2012; Hopfield, 2008; Milo et al., 2002; Watts & Strogatz, 1998). Therefore, common methods may be used to describe and understand these networks. One dynamic network analysis method, known as the community dynamic inference method (CommDy) (Berger-Wolf, Tantipathananandh, & Kempe, 2010), which is grounded in social network theory, groups nodes into communities (functional clusters) in a way that minimizes the overall changes in the community affiliations of individuals over time. CommDy was developed specifically to characterize dynamics and was originally applied to the study of social networks, which is an area of study that has been plagued by the same conceptual and computational bottlenecks seen in brain imaging data analysis. For example, CommDy has successfully characterized group behavior of sheep (Barale, Kulahci, Sulo, Berger-Wolf, & Rubenstein, 2010) and equids (Rubenstein, Sundaresan, Fischhoff, Tantipathananand, & Berger-Wolf, 2015), as well as social interactions among groups of people (Chayant Tantipathananandh, Berger-Wolf, & Kempe, 2007), although it has not yet been applied to neuroscience. A useful property of CommDy is that it allows networks to be characterized by a series of unitless metrics that correspond to the descriptions corresponding to metrics in social networks (e.g., “community size,” “visiting,” etc. See Table 1 for a listing). Such metrics provide additional information beyond more commonly applied static metrics.

**Table 1.** Descriptions of network metrics

Observed (OBS)	Number of time steps a node (pixel) was active or observed
Switching cost (SW)	Number of community switches made by an individual (normalized by the number of time steps an individual was observed)
Visiting cost (VIS)	Number of visits made by an individual to another community (normalized by the number of time steps an individual was observed)
Absence cost (ABS)	Number of absences of an individual from a community (normalized by the number of time steps an individual was observed)
Community stay (AS, MS)	Average (maximum) number of consecutive time steps an individual stayed as a member of the same community over the time steps the individual was observed
Homing (HOM)	Fraction of individual’s current community comembers, at each time step, who were comembers in the previous time step (normalized by the number of time steps an individual was observed)
Ave. group size (GS)	Average size of the groups (number of members and absents but not visitors) of which an individual was a member
Community time span (TS)	Average span of the communities (the last time step minus the first time step of the community’s existence) with which an individual was affiliated (as a member or absent)
Ave. community size (CS)	Average size of the communities (number of members and absents, but not visitors) with which an individual was affiliated (as a member or absent)

In the current report, we adapt CommDy for the analysis of flavoprotein autofluorescence imaging data from the mouse cerebral cortex in young and aged animals obtained using both brain slice and whole-animal approaches. Flavoprotein autofluorescence imaging capitalizes on intrinsic fluorescence that occurs in mitochondrial flavoproteins as neurons become active. We and others have found that such signals are stable over time and are highly sensitive to neuronal activity (D. A. Llano, Theyel, Mallik, Sherman, & Issa, 2009; Reinert, Gao, Chen, & Ebner, 2007; Shibuki et al., 2003). Here, we examine changes in the auditory and motor cortices. These brain regions were chosen based on ease of data acquisition and have both been shown to show significant vulnerability to aging (Engle & Recanzone, 2012; Hirai et al., 1996; Mendelson & Ricketts, 2001; Oliviero et al., 2006; Turner, Hughes, & Caspary, 2005; Willott, Aitkin, & McFadden, 1993). Despite our growing understanding of the changes that occur in the cortex with aging, we lack a network-level description of how aging impacts the cerebral cortex and what synaptic changes may give rise to these network-level differences. Therefore, in this study, we adapt CommDy for the analysis of neuroimaging data and compare network-level changes in the auditory and motor cortices of young and aged mice.

## RESULTS

**Figure 1.**
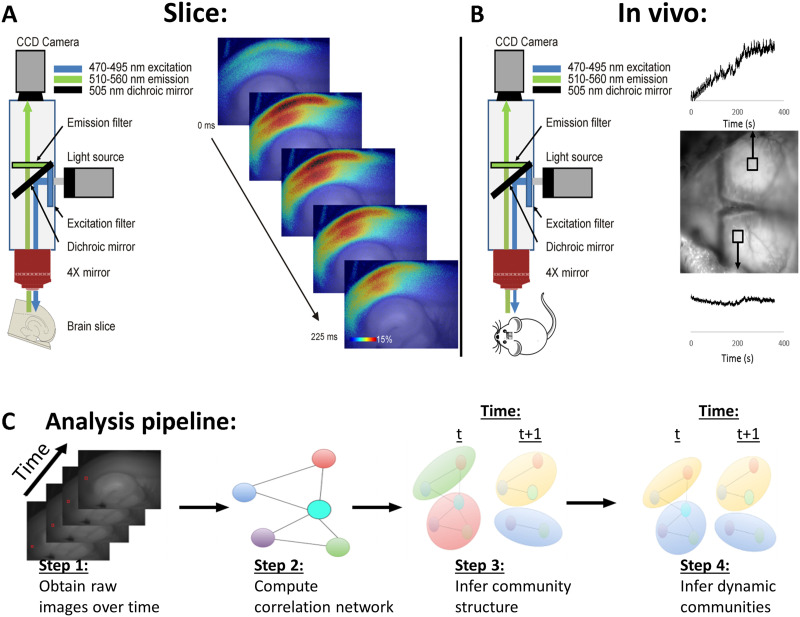
(A) Left: diagram of imaging setup for slice. The brain slice is illuminated with blue light and green light is collected using an epifluorescence setup. Right: a series of images during a paroxysmal depolarization, shown as change in fluorescence over baseline for each pixel. (B) Left: diagram of imaging setup for in vivo experiments. Right: two representative regions of interest on the surface of the mouse brain, illustrating oscillations in the raw signals. (C) Analysis pipeline showing each step of the analysis. Step 1: raw images are obtained; Step 2: time step is defined as a small (sliding) window of time, and correlation networks are computed for each time step; Step 3: the Louvain algorithm is applied to the correlation network to infer the community structure of each time step; and Step 4: CommDy is applied to the sequence of time steps of communities to infer cohesive dynamic community structure. Note that the two right-most images in C show the same temporal networks with the community labels changed from independent for each time step to cohesive over the timeline.

Two datasets were examined in this study: brain slice imaging data from the auditory cortex during paroxysmal depolarizations triggered by GABA blockade, and in vivo imaging data from the awake mouse motor cortex (see Figure 1). These regions and types of preparations were used for reasons of experimental convenience and illustrate the flexibility of the CommDy technique to handle different types of datasets. Initial development of CommDy was done on slice data, where the SNR is high, and then adapted for in vivo spontaneous activity data.

### Adaptation of CommDy for Analysis of Neural Data

Prior to applying the CommDy technique, there are several data preprocessing steps required to transform raw brain imaging data into CommDy input, including using the time series of each pixel to construct a simple network representation, and then static community detection in each time step. After that, CommDy was applied to infer dynamic communities. “Static” networks refer to temporal networks with multiple snapshots, and “dynamic” networks refer to those networks linked over time. To create the network representation, pixels were used as nodes and a weighted, thresholded network of pixel value correlations across an empirically determined number of time steps (the “window size”) was then generated. The correlation threshold was determined empirically, and correlation values above the threshold were used to build the correlation matrix.

To determine the appropriate time window and correlation thresholds for CommDy analysis applied to flavoprotein autofluorescence imaging data for slice data, the analysis window size was systematically changed from 25 frames (352 ms) to 200 frames (2,817 ms) and the resulting spatial distributions of the average degree of each node were examined (see Supporting Information Figure S1). For each analysis window (25, 50, 100, or 200 frames), the correlation coefficient for each pair of pixels was computed for the time segment where the analysis window straddled the peak of the response. The average degree of each pixel was then computed as the number of other pixels with which it had correlation coefficients in the range described above each column. The degree of each node was normalized to the maximum degree for each image. Each image, therefore, contains the spatial distribution of normalized node degree for a given analysis window size and given range of correlation coefficient. At an analysis window of 25 frames, high numbers of correlations were seen in portions of the auditory cortex at correlation coefficient thresholds between 0.7 and 0.9, but at thresholds above 0.9, spurious correlations were detected outside of the slice (dashed arrow, top right image). Using a 50-frame window, nodes restricted to the auditory cortex were seen at all thresholds above 0.7. Similar findings were seen at 100 and 200 frames. Given the increased utility of any dynamic network analysis tool at shorter analysis windows (and therefore higher temporal resolution), it was determined that the smallest window not producing spurious correlations would be used for analysis. Similar findings were seen for in vivo data (data not shown). Therefore 50 frames and a correlation threshold of 0.7 (corresponding to the red box in Supporting Information Figure S1) were the window and threshold used for all subsequent analyses.

By sliding the correlation window one step or frame each time following each correlation extraction, and doing this over the entire time line, a time series of correlation networks was obtained. CommDy can take the resulting dynamic network as the input directly or, for more efficient processing, it takes any grouping of the time steps as input. In each time step of these time series, we applied the Louvain static community inference method (Blondel, Guillaume, Lambiotte, & Lefebvre, 2008) to find snapshots of functional clusters, the “groups” that form the basis of CommDy. The Louvain algorithm was chosen because among the modern static community inference methods, its steps are highly intuitive and it is highly scalable, while achieving results comparable to other methods. The algorithm is based on two steps that are repeated iteratively to optimize the modularity in the network (Newman, 2006). First, the algorithm groups nodes into communities to maximize modularity locally. Then the algorithm hierarchically rebuilds the network, aggregating the newly discovered communities into supernodes. Thus, the nodes in this new network are communities discovered in the previous step, and the new links between those nodes are weighted by the cumulative weight of the links between the old nodes that were aggregated into the corresponding communities. The two steps are repeated iteratively until no further increase in modularity is possible.

To measure the stability of the Louvain algorithm, which is stochastic in nature, we examined six paroxysmal depolarizations in young mice and six paroxysmal depolarizations in aged mice. The Louvain algorithm was run 30 times on each paroxysmal depolarization, and we computed the standard deviation of each modularity value. We found that all standard deviations were below 5 × 10^−3^, which is lower than previous reports (Blondel et al., 2008), and, for modularity values above 0.5, the coefficient of variation was below 0.5%, suggesting that these modularity values are stable and the identified static communities are consistent across repetitions.

**Figure 2.**
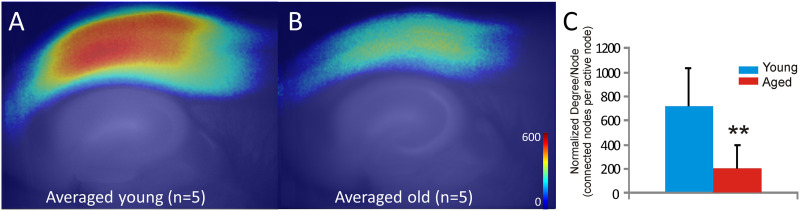
Young animals show greater average degree of each node than older animals. (A) Average normalized degree of each node, averaged across all five animals, superimposed upon an averaged anatomical map. (B) Identical analysis, but for aged animals. (C) Bar graph showing that the average degree of each node is significantly higher in the young than the aged animals. Error bars = standard deviation; **p = 0.005. See text for details.

To understand how the clusters (communities inferred by Louvain algorithm in each time step) change over time, we use the CommDy method (Berger-Wolf et al., 2010; Chayant Tantipathananandh & Berger-Wolf, 2009; Chayant Tantipathananandh et al., 2007; C. Tantipathananandh & Berger-Wolf, 2011). In CommDy, dynamic communities are essentially viewed as dynamic clusters, where the membership of the individual (represented by a node in each time step) inside the cluster is determined by the total value of the “social cost,” as manifested by the individual’s interactions over time, aiming to minimize interactions outside an individual’s community as well as switching among communities. The inferred community structure parsimoniously minimizes the overall social cost for all the individuals by using the deterministic constant-factor approximation algorithm (Chayant Tantipathananandh & Berger-Wolf, 2009). The definition of social cost is based on two explicit assumptions about individual behavior, motivated by research in social sciences and further supported by the definition of the dynamic community as a cluster. First, it assumes that individuals tend not to change their “home” community affiliation too often (Backstrom, Huttenlocher, Kleinberg, & Lan, 2006). Second, it assumes that individuals tend to interact with their respective home communities most of the time (Wasserman & Faust, 1994). These assumptions are translated into three cost parameters potentially incurred by an individual. First, the CommDy method posits a cost for a switch from one community to another. Second, there is a cost of visiting a community of which one is not a member. Third, in datasets for which not all individuals are observed all the time, there is a cost of absence for an individual who is not observed at a gathering of a community of which it is a member. A dynamic community is then defined as a time series of sets of individuals among whom the overall social cost of interacting is minimized (Berger-Wolf et al., 2010). Note that these costs have not yet been defined for brain networks and were set to equal values for our initial studies, with subsequent exploration of the range of the parameters. For visualization, once communities were assigned, the pixels belonging to the top 20 communities over the entire time line were distinguished using a palette of 20 colors (Figure 2).

CommDy can be used to quantitatively describe network activity in terms of the node and structural network metrics based on network theory (Rubenstein et al., 2015). These metrics permit quantitative comparisons between different networks (e.g., comparing the cortical networks in aged vs. young animals). Therefore, quantitative analyses of CommDy activation patterns were performed using 10 relevant community metrics that describe the interactions between pixels (see Table 1 for a listing and definition of the metrics).

### Impact of Aging on Network Dynamics in Brain Slice Preparations

Paroxysmal depolarizations were imaged in brain slices taken from five young (all were 5.5 months old) and five aged (all were 22 months old) animals (a total of 41 and 30 activations, respectively). Initially, static analysis was performed on auditory cortical networks in young and aging mice. To do this, the average degree of each node was computed for each pixel as the average number of edges at each node during the 100-frame window surrounding the peak of the paroxysmal depolarization. These values were then averaged across paroxysmal depolarizations within each animal, normalized to the overall magnitude of the response, and the resulting grand average for each group of animals is shown in Figure 2. As shown, the average normalized degree of each node was substantially higher in younger animals than in aged animals (mean normalized node degree in young animals = 813.0 ± 353.6 [*SD*] edges per active node, mean in aged animals = 149.1 ± 162.4 [*SD*] edges per active node, *p* = 0.005, Mann–Whitney). These data suggest that the average connectivity between nodes is higher in younger animals than in aging animals and cannot be accounted for by any differences in the magnitude in the response of the young versus aged animals.

**Figure 3.**
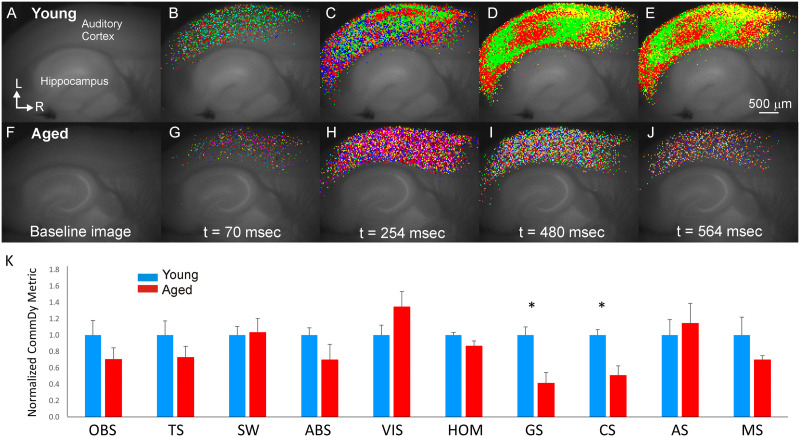
Representative CommDy population dynamics across time for a paroxysmal depolarization in a young animal (A–E) and an aged animal (F–J). Each color represents a different community. (K) Bar graph of average CommDy metrics for young (*n* = 40 activations) and aged (*n* = 31 activations) animals, normalized to the average value for young animals. Group size, community size, average stay, and maximum stay are normalized for the total number of activated pixels. OBS = observed; TS = community time span; SW = switching cost; ABS = absence cost; VIS = visiting cost; HOM = homing; GS = average group size; CS = average community size; AS = average community stay; MS = maximum community stay. Fuller explanation of these metrics is found in Table 1. Error bars = standard error; *p < 0.05.

Average degree of each node does not provide insights into the changing associations between nodes. We therefore performed dynamic analysis using CommDy, which is built upon static communities detected by the Louvain algorithm independently in each time step. Dynamic analysis of paroxysmal depolarizations in young and aged animals using CommDy revealed that network activity differed between these two groups. Young animals showed distinct patterns of activity across the auditory cortex during paroxysmal depolarizations. See Figure 3A–3E for an example from one representative young mouse. Early during the response, multiple small clusters (communities) were observed (Figure 3B). As the response evolves, a distinct cluster (colored red) emerges in the upper layers of the auditory cortex (Figure 3C), which then spreads to produce two broad red networks – one in the upper layers and another in the lower layers – which persists to at least 500 msec after onset (Figure 3E; see Supporting Information Movie 1). In the aged animals, there is also broad activation across the auditory cortex. However, the activation is much more heterogeneous than that seen in the young animal and comprises nodes of many different communities and never coalesces into coherent clusters seen in the young animal (see Figure 3F–3J for a representative aged mouse; see Supporting Information Movie 2).

CommDy network metrics were compared between young and aged mice. To ensure that differences in network measures were not due to changes in the absolute magnitude of activation, individual group and community sizes were normalized by the total number of nodes in each activation. Using a mixed-effects model, age was found to be a significant predictor for both normalized group size (mean values = 0.211 ± 0.113 [*SD*] for young vs. 0.058 ± 0.046 [*SD*] for aged; *p* = 0.0001) and normalized community size (mean values = 0.235 ± 0.142 [*SD*] for young vs. 0.046 ± 0.044 [*SD*] for aged; *p* = 0.0002). The differences in homing, a measure of network cohesion, trended toward significance but did not survive correction for multiple comparisons (mean values = 0.756 ± 0.663 [*SD*] for young vs. 0.125 ± 0.081 [*SD*] for aged; *p* = 0.0168). See Figure 3K for a comparison of all 10 network metrics.

### Impact of Hearing Loss and Cortical Thickness on CommDy Parameters

Age, cortical thinning and hearing loss are known to be correlated (Ha et al., 2020; Li et al., 2012; Salat et al., 2004; Stebbings et al., 2016). Therefore, to disentangle these influences on cortical network activity, we asked whether the aging-related changes in group and community size could be better explained by cortical thickness or hearing loss. We first measured the differences in hearing threshold between the groups, and, as expected, differences in aged and young animals were noted in hearing thresholds (33.9 ± 2.2 [*SD*] dB SPL [sound pressure level] for young and 59.9 ± 27.6 [*SD*] dB SPL for aged, *p* = 0.032, Mann–Whitney; Supporting Information Figure S2A). Activations were examined from animals with high auditory brainstem response (ABR) thresholds (≥45 dB SPL, *n* = 3 mice) and compared to those with low ABR thresholds (<45 dB SPL, *n* = 7 mice). Similar to separation of groups based on age (*n* = 5 per group), using a mixed-effects model, normalized group size, and normalized community size were both larger in the better hearing animals. The values for normalized group size = 0.189 ± 0.070 (*SD*) for low ABR threshold versus 0.120 ± 0.052 (*SD*) for high ABR threshold, (*p* = 0.0002, Supporting Information Figure S2B). The values for normalized community size = 0.215 ± 0.058 (*SD*) for young vs. 0.146 ± 0.052 (*SD*) for aged (*p* = 0.0001, Supporting Information Figure S2C). In addition, as previously described in a related dataset (Stebbings et al., 2016), we observed differences in cortical thickness between the two groups (1.20 ± 0.04 [*SD*] mm for young and 1.04 ± 0.04 [*SD*] mm for aged, *p* = 0.012, Mann–Whitney; Supporting Information Figure S3A). Partial correlations between cortical thickness and group size as well as between cortical thickness and community size were computed. Partial correlations were used to account for the impact of different numbers of activations in different animals. Significant correlations were observed in both cortical thickness versus group size (*r* = 0.734, *p* < 0.0001; Supporting Information Figure S3B) and cortical thickness versus community size (*r* = 0.722, *p* < 0.0001; Supporting Information Figure S3C). Given the multiple potential related predictors (age, hearing loss, cortical thickness) of group and community size, a linear mixed model multivariate regression was conducted using all three predictors. For community size, age was the only significant predictor (*p* = 0.011), whereas for group size, cortical thickness was the only significant predictor (*p* = 0.046; see Supporting Information Table S1).

### Aging-Related Changes Are Related to Declines in Intracortical Connectivity

**Figure 4.**
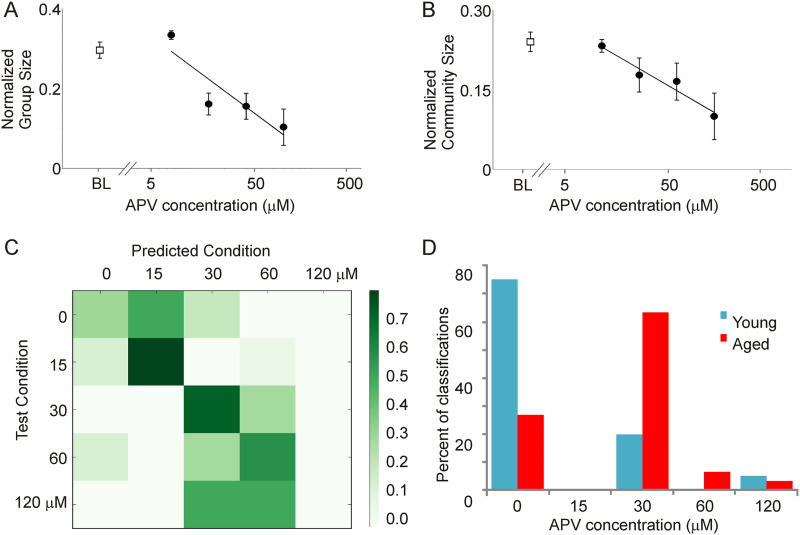
Impact of increasing concentrations of bath-applied APV at concentrations of 15 (*n* = 14 activations), 30 (*n* = 9), 60 (*n* = 9), and 120 μM (*n* = 5) on normalized group size (A) and normalized community size (B) in a coronal slice preparation from a young adult mouse. BL = baseline values prior to adding APV (*n* = 10). (C) Confusion matrix showing the performance of the random forest classifier. True categories are shown in the rows, and predicted category is shown in the columns. Most data are seen on or near the diagonal, yielding an accuracy of 0.575. (D) Using the same model to classify data from young versus aged mice, most activations from young animals (30/40) fall correctly into the baseline (no drug) category, while most of the activations from aged animals (22/30) fall into a drug category.

To test whether the aging-related changes in group and community size described above may be related to changes in intracortical connectivity, diminished intracortical connectivity was pharmacologically simulated in a coronal brain slice from a young animal by blocking NMDA receptors, which are enriched in cortico-cortical synapses (Fleidervish, Binshtok, & Gutnick, 1998; Gil, Connors, & Amitai, 1999). Unlike the thalamocortical slice described above, this preparation does not contain connectivity between subcortical nuclei and the cortex and thus provides an indicator of the impact of intracortical connections. A concentration-dependent drop in both normalized group and community size was seen with bath application of the NMDA blocker APV (1-way ANOVA, *p* = 5 × 10^−9^ for group size, *p* = 0.004 for community size; Figure 4A and 4B). To further determine whether network changes observed during NMDA blockade resembled network changes that occur during aging, a classifier was built using the averaged metrics from Table 1 obtained for the NMDA blockade data as input features, and the ability of this classifier to distinguish patterns of activity in young versus aged was measured. A random forest method (Breiman, 2001) was used to build a classifier by using the five different classes of NMDA blockade data (baseline, 15, 30, 60, and 120 μM APV). Given the five dose categories, chance performance of the classifier = 0.2 (or 0.35 using the majority class as the default prediction). Using 50 trees and leave-one-out validation, the accuracy of the classifier for APV data = 0.575, which is greater than that expected by chance (see confusion matrix, Figure 4C). When applied to all data obtained from young and aged animals, this classifier identified 30 out of 40 young as belonging to the baseline group and 22 out of 30 aged as belonging to one of the APV groups, thus “correctly” identifying 74% of the activations (Figure 4D). When comparing the 30-μM dose group (the category into which most of the aged data fell) to the baseline group, the overall accuracy of the model was 0.70. Thus, using entirely different datasets for model development and model validation, these data suggest that the aged data, when looked at from a network perspective, bear strong commonalities to the effect of bathing a young slice in 30 μM APV. These data also suggest that more complete blockade, as is present with 120 μM APV, does not replicate aging, suggesting that aging is associated with a partial loss of cortico-cortical connectivity.

### Impact of Aging on Network Dynamics In Vivo

**Figure 5.**
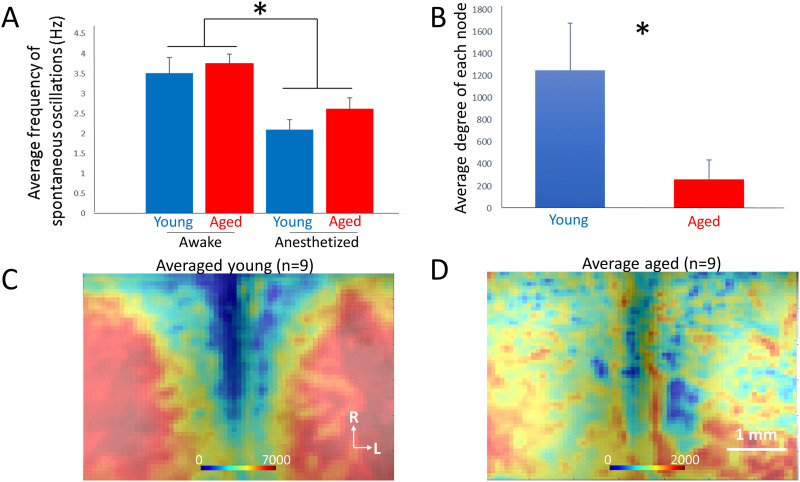
(A) The average frequency of spontaneous activity was roughly 3.5 Hz in awake mice and similar between aged and young mice. The average frequency declined significantly to approximately 2.5 Hz in the presence of ketamine/xylazine anesthesia. (B) Comparison of average degree per node (measured as number of edges per node) in young versus aged mice (*n* = 9 per group). (C) Average map of degree of each node of *n* = 9 young mice. (D) Average map of degree of each node of *n* = 9 aged mice. In both C and D, heat map corresponds to the number of edges of each node; *p < 0.05.

Nine young (average age = 4.7 months, range = 4.4 to 5.0 months) and nine aged (average age = 23.2 months, range = 23.0 to 23.5 months) mice were used for these experiments. Because flavoprotein autofluorescence has not been used previously to measure spontaneous neural activity in vivo, we initially characterized the properties of the signals. It was observed that the majority of the power is in the delta range (approximately 3 Hz) in both young and aged animals (Figure 5A). To validate that the spectral properties are modulated as expected by level of arousal, mice were anesthetized with ketamine/xylazine (after spontaneous signals were measured for CommDy analysis) and we observed the expected overall decrease in frequency in both groups (*p* = 0.006), as has been seen previously (Dworak, McCarley, Kim, & Basheer, 2011; Schüttler et al., 1987), with no differences between age groups (*p* = 0.3, Figure 5A). To determine the robustness of the spontaneous flavoprotein oscillatory signals in young compared to aged mice, the SNR of the spontaneous activity was compared and found to be similar in the two groups (average young SNR = 1.64 [*SD* 5.1] dB, average aged SNR = 1.99 [*SD* 4.34] dB, *p* = 0.79, Mann–Whitney). These data suggest that spontaneous flavoprotein autofluorescence signals track with more commonly measured indicators of spontaneous activity, such as EEG, and show similar oscillatory properties at in young and aged mice, but given the slow nature of the signal are limited to lower frequency spectral bands than EEG.

**Figure 6.**
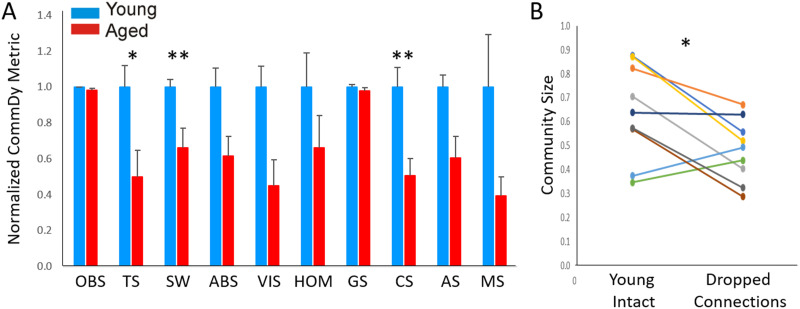
(A) Bar graph of average CommDy metrics for spontaneous in vivo data young (*n* = 9) and aged (*n* = 9) animals, normalized to the average value for young animals. Group size, community size, average stay, and maximum stay are normalized for the total number of activated pixels. Error bars = standard error. (B) Comparison of community size (a unitless value) in young mice either before (left) or after (right) randomly dropping edges so that the number of edges equaled the number of edges seen in aged animals. Each line corresponds to a particular animal before and after dropping connections. OBS = observed; TS = community time span; SW = switching cost; ABS = absence cost; VIS = visiting cost; HOM = homing; GS = average group size; CS = average community size; AS = average community stay; MS = maximum community stay. Fuller explanation of these metrics is found in Table 1; *p < 0.05, **p < 0.01.

To compare the overall degree of connectedness between nodes in aged versus young animals, the degree of each node was computed in young and aged animals. Similar to the findings in brain slices from the auditory cortex, the degree of connectivity, as measured by average number of edges per node, was significantly decreased in the aged compared to the young animals (1,250 ± 1,214 [*SD*] edges/node vs. 257 ± 476 [*SD*] edges/node, *p* = 0.03, Mann–Whitney; Figure 5B). When plotted as average 2-D maps, in young animals there are large regions with high connectivity that are seen symmetrically on either side of the midline (Figure 6C). In contrast, 2-D maps in older animals show essentially no organization, with scattered islands of connectivity with rough symmetry across the midline (Figure 5D).

Characterization of network structure using CommDy revealed aged versus young differences for three different network metrics: time span (young = 0.686 ± 0.229 [*SD*], aged = 0.342 ± 0.289 [*SD*], *p* = 0.014, Mann–Whitney), switching (young = 0.920 ± 0.111 [*SD*], aged = 0.609 ± 0.283, *p* = 0.006, Mann–Whitney), and normalized community size (young = 0.642 ± 0.198 [*SD*], aged = 0.325 ± 0.173 [*SD*], *p* = 0.0062, Mann–Whitney; Figure 6A). Several other network metrics also showed differences that trended toward significance: absence (young = 0.323 ± 0.098 [*SD*], aged = 0.199 ± 0.099 [*SD*], *p* = 0.027, Mann–Whitney), visiting (young = 0.256 ± 0.083 [*SD*], aged = 0.115 ± 0.105 [*SD*], *p* = 0.017, Mann–Whitney), and average stay (young = 0.680 ± 0.128 [*SD*], aged = 0.411 ± 0.231 [*SD*], *p* = 0.017, Mann–Whitney); but these did not survive multiple comparisons correction. To determine if the drop in community size with aging is related to diminished intracortical connectivity, analogous to that seen in vitro (see Figure 4B), connections between nodes were randomly dropped so that the average degree per node in the young mice matched the aged mice and network metrics were recomputed. This manipulation was intended to mimic the NMDA blockade done in vitro. Similar to the findings in vitro, community size was found to decline with decreasing connectivity between nodes (young intact community size = 0.642 ± 0.198 [*SD*], young after dropped connections community size = 0.480 ± 0.130 [*SD*], *p* = 0.03, Wilcoxon signed rank test; Figure 6B). These data suggest that in both datasets (slice and in vivo), the effect of aging is to decrease cortical network community size by diminishing intracortical connectivity.

## DISCUSSION

In this study, we applied the CommDy network analysis algorithm to investigate changes in dynamic network structure that occur in cortical networks as they age. After optimizing CommDy to detect communities in a brain slice preparation, CommDy was used to compute a series of metrics that reflect the “social” organization of the cortical networks. The common finding between brain slice paroxysmal activations and in vivo awake spontaneous activity was a decline in both degree of each node and cortical network community size with aging. Community size is a reflection of temporary associations between nodes in a network. The decline in community size seen with aging was mimicked by weakening cortical connections pharmacologically (with the NMDA blocker APV; Figure 4) or computationally (by randomly dropping edges; Figure 6), suggesting that aging is associated with weakening of intracortical connections. Given the differences in the two datasets used in this study (slice vs. in vivo, auditory vs. motor cortex, paroxysmal activity vs. routine spontaneous activity), it is remarkable that the data converged to show diminished community size with aging. Below we describe the limitations and the potential utilities of the CommDy technique in brain imaging studies and how they relate to changes occurring during aging.

### Technical Considerations

The current work is based on flavoprotein autofluorescence imaging, which has the advantages of providing a stable intrinsic signal with high sensitivity and good spatial resolution (100–200 microns; D. A. Llano et al., 2009), but has a relatively sluggish time course (∼0.5–1 s for time to peak; D. A. Llano et al., 2009; Reinert et al., 2007; Shibuki et al., 2003). Given this slow time course, one might expect that small differences in the time to activate of different groups of neurons would be washed out by delays induced by neurometabolic coupling. However, despite these slow responses, CommDy did reveal distinct populations, suggesting that more subtle heterogeneities than could be appreciated using traditional analytical approaches were detectable using CommDy in the time courses of the imaging responses. Another potential methodological consideration about the current analysis is the use of pixels as individual nodes, rather than using defined regions or individual neurons. At the binning and magnification used in this study, an individual pixel occupies an area of approximately 800–1,600 μm^2^. To estimate the number of neurons represented in this pixel, an upper estimate of the volume will be used, corresponding to the full thickness of the slice or the cortex, though it is likely that the blue excitation light incompletely penetrated the sample. The mouse cerebral cortex contains about 9.2 × 10^4^ neurons/mm^3^ (Schüz & Palm, 1989), giving a rough estimate that each pixel in our preparation contained signal emanating from on the order of ∼50–100 neurons. Compared to techniques that image spiking activity of individual cells, such as calcium imaging, flavoprotein autofluorescence offers lower spatial resolution but offers a broad field of view (multiple millimeters), permitting large-scale networks to be identified. Compared to the most commonly used technique for large-scale brain imaging, fMRI with BOLD signals, where the voxel sizes range from millimeters in humans to hundreds of microns in rats, the current analysis provides substantially higher spatial resolution. In addition, we have recently applied CommDy to calcium imaging data from cortical slices and observed that CommDy is able to detect known features of cortical connectivity (Naik et al., 2019). Therefore, it is likely that the current analytical approach may be adapted to any of the imaging techniques mentioned above.

### Implications for Aging

Pathological changes associated with aging have been described at virtually every level of the central nervous system, including the cortex. Here, we observed aging-related disruptions in network-level function of the auditory and motor cortices. In the slice preparation, these changes are independent of the magnitude of the activation and are likely not related to differences in slice viability, since we have previously found that the auditory cortices in slices from young versus aged animals do not differ in their baseline redox state or response to metabolic stress (Stebbings et al., 2016). We note that these data are not likely to be explained by any potential viability differences in the preparations because simple loss of connectivity does not by itself directly imply a lack of cohesion over time, as the small groups can still coalesce into functional units. For example, even at low densities, the presence of well-connected clusters has been shown to result in synchronization in static networks (Du & Li, 2014; Ponce-Alvarez et al., 2015; Zhao, Bryce Beverlin, Netoff, & Nykamp, 2011). In addition, differences between young and aged animals in the brain slice studies are also likely not related to differences in GABAergic synaptic inhibition, since saturating doses of a GABA_A_ antagonist were used in the slice study.

The current data suggest that aging causes changes in the underlying local excitatory substructure in the cerebral cortex (Figures 4 and 6). The specific substrate for these changes is unknown, but could relate to changes in local dendritic branching and integration, changes in intrinsic excitability of pyramidal cells, or changes in synaptic properties, all known to be altered in the aging brain (Cupp & Uemura, 1980; Disterhoft & Oh, 2007; Feldman & Dowd, 1975; Leuba, 1983). Regarding the latter hypothesis, we observed that a classifier built only using dose-dependent changes in CommDy network metrics induced by APV was able to classify data from young versus aged animals with a high degree of accuracy (Figure 4). These findings are remarkable in that the cross-validation was done using entirely different datasets obtained from different types of slices (auditory cortex slices for aging data and coronal slice for APV data). Furthermore, these findings are consistent with a body of literature demonstrating NMDA receptor hypofunction in the cerebral cortex of the aging brain (Magnusson & Cotman, 1993; Piggott, Perry, Perry, & Court, 1992; Wenk, Walker, Price, & Cork, 1991). Therefore, these data suggest that the aging-related changes in network-level interactions observed in the cortex of this and previous studies (Andrews-Hanna et al., 2007; Battaglia et al., 2020; Tomasi & Volkow, 2012) may be, at least in part, caused by changes in NMDA receptor function.

Hearing loss and cortical thickness were both associated with declines in group and community size in aging auditory cortical networks (Supporting Information Figures S2 and S3). Hearing threshold is known to increase with age in mice, including the CBA/CaJ mice studied here (Zheng, Johnson, & Erway, 1999). The relationship between hearing loss and auditory cortical network structure in the current data is likely confounded by age as the relationships between hearing loss and community and group size were found not to be significant in a multivariate regression when age and cortical thickness were included as predictors. However, cortical thickness was also found to be an independent predictor of normalized group size (Supporting Information Table S1). This relationship is consistent with the mechanisms of aging-associated thinning of the cortex. Cortical thinning is related to the loss of synapses between cortical neurons and/or demyelination of local cortical axons, with retention of the number of neurons (Dumitriu et al., 2010; Haug, Kühl, Mecke, Sass, & Wasner, 1983; Masliah et al., 1993; Terry, DeTeresa, & Hansen, 1987). As such, cortical thinning would be predicted to be associated with drops in cortical connectivity, consistent with the current data. This idea is supported by previous findings from network theory showing that random or uniform loss of connectivity, translated into uniformly lower network density, results in smaller clusters (Erdos & Rényi, 1959, 1960). Hence, our results showing diminished group and community size with aging, combined with the known drop in synaptic connectivity seen in aging, are consistent with predictions from network theory.

### Potential Utilities of CommDy

The notion that neuronal cell assemblies are critical for perception and may shift dynamically over time is a foundational principle in neuroscience (Abeles, Prut, Bergman, & Vaadia, 1994; Singer, 1998), but quantifying such network dynamics has been challenging. CommDy may prove to be a useful tool to quantitatively study brain networks. Although the current study is focused on flavoprotein autofluorescence imaging, CommDy can be adapted to other forms of brain imaging as well. Such adaptation would permit CommDy to be used to refine network-level hypotheses about brain function, for example, in the study of pathological states in humans. It is speculated that many neurological and psychiatric disorders are caused by functional disruptions in large-scale brain networks (Filippi et al., 2012; Rubinov et al., 2009; Seeley, Crawford, Zhou, Miller, & Greicius, 2009). Most of the current work in this area has focused on using network measures either as a tool to classify different disease states, or as a means to better understand network disruptions associated with disease states. In both cases, much of the work has been focused on the generation of static maps. However, in brain diseases, fluctuations in clinical symptomatology are the rule rather than the exception. For example, seizures in epilepsy, “off” states in Parkinson’s disease, and hallucinations in schizophrenia all occur paroxysmally on the backdrop of stable brain structures, such that it will only be possible to understand them using dynamic assessment tools. CommDy may add to the growing toolbox of network assessment tools and will contribute to the understanding of brain network dynamics.

## METHODS

### Animal Use

CBA/CaJ mice from 4.4 to 23.5 months of age of both sexes were used. All procedures were approved by the Institutional Animal Care and Use Committee at the University of Illinois. All animals were housed in animal care facilities approved by the American Association for Assessment and Accreditation of Laboratory Animal Care.

### Auditory Brainstem Responses

As previously described (Stebbings et al., 2016), to measure hearing for the aging studies, ABRs were obtained in response to tones at frequencies of 8, 16, and 32 kHz, and average thresholds were reported. Animals were anesthetized with 100 mg/kg ketamine + 3 mg/kg xylazine intraperitoneally before the insertion of two subdermal electrodes, one at the vertex and one behind the left ear. Stimuli were presented using a Tucker-Davis (TDT) system 3, ES1 free field speaker, with waveforms being generated by RPvdsEx software. The output of the TDT speaker was calibrated at all the relevant frequencies, using a Bruel and Kjaer type 4135 microphone and a Bruel and Kjaer measuring amplifier (Model 2610). Each frequency was presented for 5 ms (3 ms flat with 1 ms for both rise and fall times), at a rate of 2–6 Hz with a 100 ms analysis window. Raw potentials were obtained with a Dagan 2400A amplifier and preamplifier headstage combination, and filtered between 100 Hz and 3000 Hz. An ADInstruments PowerLab 4/30 system was used to average these waveforms 500 times. Significant deflections, assessed via visual inspection, within 10 ms after the end of the stimulus were deemed to be a response. Blinding of ABR assessments was not done given the conspicuous physical differences in young versus aged mice (i.e., aged animals are heavier and had less hair on snout).

### Brain Slice Preparation

The CBA/CaJ strain was used because of its gradual aging-related hearing loss (Frisina & Zhu, 2010; Ohlemiller, Dahl, & Gagnon, 2010; Zheng et al., 1999). Five young mice (5.5 months of age) and five aged mice (22 months of age) were used. Mice were initially anesthetized with ketamine (100 mg/kg) and xylazine (3 mg/kg) and then transcardially perfused with an ice-cold sucrose saline solution (in mM: 206 sucrose, 10.0 MgCl_2_, 11.0 glucose, 1.25 NaH_2_PO_4_, 26 NaHCO_3_, 0.5 CaCl_2_, 2.5 KCl, pH 7.4). Slices containing the auditory cortex were cut using a modification of the method developed by Cruikshank et al. (Cruikshank, Rose, & Metherate, 2002), modified for the aging brain, as described previously (Stebbings et al., 2016; Takesian, Kotak, & Sanes, 2012), see Figure 1B for brain image. Brains were blocked by removing the olfactory bulbs and the anterior 2 mm of frontal cortex with a razor blade. The brain was then tipped onto the coronal cut and an off-horizontal cut was made on the dorsal surface, removing a sliver of brain angled at 20° from the horizontal plane. The brain was then glued onto the cut angled surface, and sections were then taken. All slices were then transferred to a holding chamber containing oxygenated incubation artificial cerebrospinal fluid (ACSF) (in mM: 126 NaCl, 3.0 MgCl_2_, 10.0 glucose, 1.25 NaH_2_PO_4_, 26 NaHCO_3_, 1.0 CaCl_2_, 2.5 KCl, pH 7.4) and incubated at 32°C for 1 hr prior to experimentation. The ACSF used for the experiments use equimolar MgCl_2_ and CaCl_2_ and contained 1 μM of the GABA_A_ antagonist SR95531 to enhance spontaneous activity. Without the addition of SR95531, we found that the amount of activity present in the slice was insufficient for either CommDy or traditional analyses. During imaging, slices were placed on a stainless steel mesh for two-sided perfusion, as we have described previously (Daniel A. Llano, Slater, Lesicko, & Stebbings, 2014). To assess the impact of NMDA blockade on network activity, the NMDA blocker D-APV (Tocris, catalog no. 0106) was dissolved in ACSF and bath applied at a series of concentrations ranging from 15 to 120 μM, with 20-min wash-ins between each dose escalation. Cortical thickness was evaluated by drawing a line tangent to the rostral-most extent of the hippocampus, from the white/gray matter border of the cortex to the pia, as we have described previously (Stebbings et al., 2016).

### In Vivo Preparation

An initial stereotactic surgery under ketamine/xylazine (100 mg/kg and 3 mg/kg IP, respectively) was done to glue a threaded headbolt onto the skull using OptiBond XTR kit (catalog no. 35106) cement. After at least 3 days to recover, mice were gradually acclimated to an imaging chamber within a soundproof booth. Their headbolts were affixed to a holder, and the body was suspended while under isoflurane (4%) anesthesia. The mice were then allowed to emerge from anesthesia, initially for 5 min, and gradually up to 15–20 min. Awake data were obtained once isoflurane was removed for at least 10 min. Once awake data were obtained, the animal was reanesthetized with ketamine/xylazine (100 mg/kg and 3 mg/kg IP, respectively) for additional imaging.

### Imaging

For the slice work, flavoprotein autofluorescence imaging was done with a fluorescence illuminator (Prior Lumen 200) and a UMNIB Olympus filter cube (470–490 nm excitation, 505 nm dichroic, 515 nm emission long pass). A coverslip was placed over the slice to provide a stable imaging plane, and data were collected using an infinity-corrected 4X macro objective (NA 0.28) and a Retiga EXi camera and StreamPix software (see Figure 1A for diagram of experimental setup). Data were obtained with 8 × 8 hardware binning, producing images of 130 × 174 pixels. Acquisition rates were 71 frames per second and 1,000 frames were used for analysis.

In vivo imaging was done using a macroscope system outfitted with 85 mm f/1.4 and f/1.2 Nikon lenses and an Adimec 1,000 m CCD camera (7.4 × 7.4 μm pixel size, 1,004 × 1,004 pixels). Blue light (450 nm, 30 nm band-pass) was used for excitation and green light (515 nm, long pass) was collected, and a 495-nm dichroic mirror was used. Images were collected at 25 frames per second. One thousand frames were used for the analysis. Pixels were binned 4 × 4, and a region of interest containing 60 × 70 pixels over both motor cortices and symmetric with respect to the midline was selected for analysis. No filtering was done on the signals. SNR was computed using the MATLAB function snr on the time series obtained by taking the time course of the average of all pixels in the image.

### Paroxysmal Depolarizations

In the presence of SR95531, spontaneous activations were observed with flavoprotein autofluorescence imaging, consisting of a relatively sharp rise in fluorescence, peaking at about 150 ms after baseline, with a slow decline, gradually returning to baseline seconds. We routinely observe these spontaneous activations in all brain slices that contain the cerebral cortex and are bathed in concentrations of SR95531 above 0.5 μM, and generally such activations occur roughly every 30–60 s with no obvious periodicity. These electrical events strongly resemble the intra- and extracellular manifestations of paroxysmal depolarizing shifts, respectively (Johnston & Brown, 1981; Steriade & Amzica, 1999; Vanzetta, Flynn, Ivanov, Bernard, & Bénar, 2010; Yaron-Jakoubovitch, Koch, Segev, & Yarom, 2013), and we have previously found that they correlate with paroxysmal depolarizing shifts (Ibrahim et al., 2017). Therefore, we refer to them as paroxysmal depolarizations. All slice analyses in this report are done on activity occurring during the paroxysmal depolarizations.

### Statistical Analysis

To compare activations in slices from young versus aged mice, a linear mixed model analysis was run, incorporating random effects because multiple paroxysmal depolarizations occurred in some slices. The 10 CommDy network metrics were used As regression variables and young versus aged was used as treatment groups. Other analysis was done without the assumption of normality, thus Mann–Whitney testing was used to compare groups, Spearman’s correlations were used for bivariate data, and Wilcoxon testing was used for paired data. The Holm–Bonferroni method (Holm, 1979) was used to adjust for multiple comparisons.

## ACKNOWLEDGMENTS

The authors thank David Trinco, Syed Haider and the University of Illinois Statistical Consultation Service for their assistance with data analysis.

## SUPPORTING INFORMATION

Supporting information for this article is available at https://doi.org/10.1162/netn_a_00191.

## AUTHOR CONTRIBUTIONS

Daniel Adolfo Llano: Conceptualization; Data curation; Formal analysis; Funding acquisition; Investigation; Methodology; Project administration; Resources; Supervision; Validation; Writing – original draft; Writing – review & editing. Chihua Ma: Conceptualization; Formal analysis; Investigation; Methodology; Writing – original draft; Writing – review & editing. Umberto Di Fabrizio: Conceptualization; Formal analysis; Investigation; Methodology; Writing – original draft; Writing – review & editing. Aynaz Taheri: Conceptualization; Formal analysis; Investigation; Methodology. Kevin A. Stebbings: Investigation; Methodology. Georgiy Yudintsev: Data curation; Investigation; Methodology. Gang Xiao: Formal analysis. Robert V. Kenyon: Conceptualization; Funding acquisition; Investigation; Methodology; Project administration; Visualization; Writing – original draft; Writing – review & editing. Tanya Y. Berger-Wolf: Conceptualization; Data curation; Formal analysis; Funding acquisition; Investigation; Methodology; Project administration; Resources; Software; Supervision; Visualization; Writing – original draft; Writing – review & editing.

## FUNDING INFORMATION

Daniel Adolfo Llano, National Science Foundation (https://dx.doi.org/10.13039/100000001), Award ID: 1515587. Daniel Adolfo Llano, National Institutes of Health (https://dx.doi.org/10.13039/100000002), Award ID: DC012125. Daniel Adolfo Llano, National Institutes of Health (https://dx.doi.org/10.13039/100000002), Award ID: AG059103. Daniel Adolfo Llano, American Federation for Aging Research (https://dx.doi.org/10.13039/100000965), Award ID: 2011-03147. Daniel Adolfo Llano, Alzheimer's Association (https://dx.doi.org/10.13039/100000957), Award ID: NIRG-12-242848. Daniel Adolfo Llano, Kiwanis Neuroscience Research Foundation.

## TECHNICAL TERMS

Flavoprotein autofluorescence: An imaging method that uses changes in the redox state of mitochondria as an indicator of neuronal activity. Activated mitochondria fluoresce green when stimulated with blue light.
Paroxysmal depolarization: A change in voltage in a neuron that represents a brief period of extreme excitability. These depolarizations are typically seen during seizures or events that predict the later development of seizures.
GABA: Gamma-aminobutyric acid, which is the main inhibitory neurotransmitter in the brain.
Louvain: A method to detect groups of functionally-connected nodes based on optimization of modularity.
Degree: The number of other nodes with which one node shares a functional connection, as determined by correlation coefficient.
Auditory brainstem response: A surface electrophysiological tool to measure peripheral hearing. When sounds reach the brain, they cause a series of small voltage deflections that can be detected with a surface electrode, and the minimal sound amplitude that elicits this response is known as the hearing threshold.
NMDA Receptor: A subtype of glutamate receptor that is in part responsible for excitatory connections between neurons and is involved when synaptic strength changes over time.
APV: A drug that blocks NMDA receptors.
EEG: Electroencephalography, which is a technique to measure electrical activity of the brain by placing electrodes on the scalp.
CBA/CaJ: A mouse strain commonly used in auditory aging studies because it retains relatively good hearing until older ages.
Artificial cerebrospinal fluid: A laboratory solution used to mimic the fluid that normally surrounds the brain.
SR95531: A compound used to block GABA receptors in the brain. Blocking these receptors leads to hyperexcitability in the tissue.
Hippocampus: A brain region important for learning and memory. In this case, the hippocampus serves as a landmark for the location of the auditory cortex.
Dichroic: A mirror that reflects certain wavelengths of light, but not all, used to direct light in a fluorescence imaging system.
Macroscope: An imaging system using tandem photography lenses used to maximize brightness of signal using wide-field imaging at relatively low magnifications.
