# Supplementary material for: A novel dynamic network imaging analysis method reveals aging-related fragmentation of cortical networks in mouse
Source: Netw Neurosci. 2021 Jun 21;5(2):569–90. doi: 10.1162/netn_a_00191 (PMC8233117; doi:10.1162/netn_a_00191)
Supplement: Supplementary file 1 [file netn-05-569-s001.pdf]

Llano, D. A, Ma, C., Di Fabrizio, U., Taheri, A., Stebbings, K. A., Yudintsev, G., Xiao, G., Kenyon, R. V., & Berger-Wolf, T. Y. (2021). Supporting information for "A Novel dynamic network imaging analysis method reveals aging-related fragmentation of cortical networks in mouse." *Network Neuroscience.* Advance publication. https://doi.org/10.1162/netn_a_00191

Supplemental Figures:

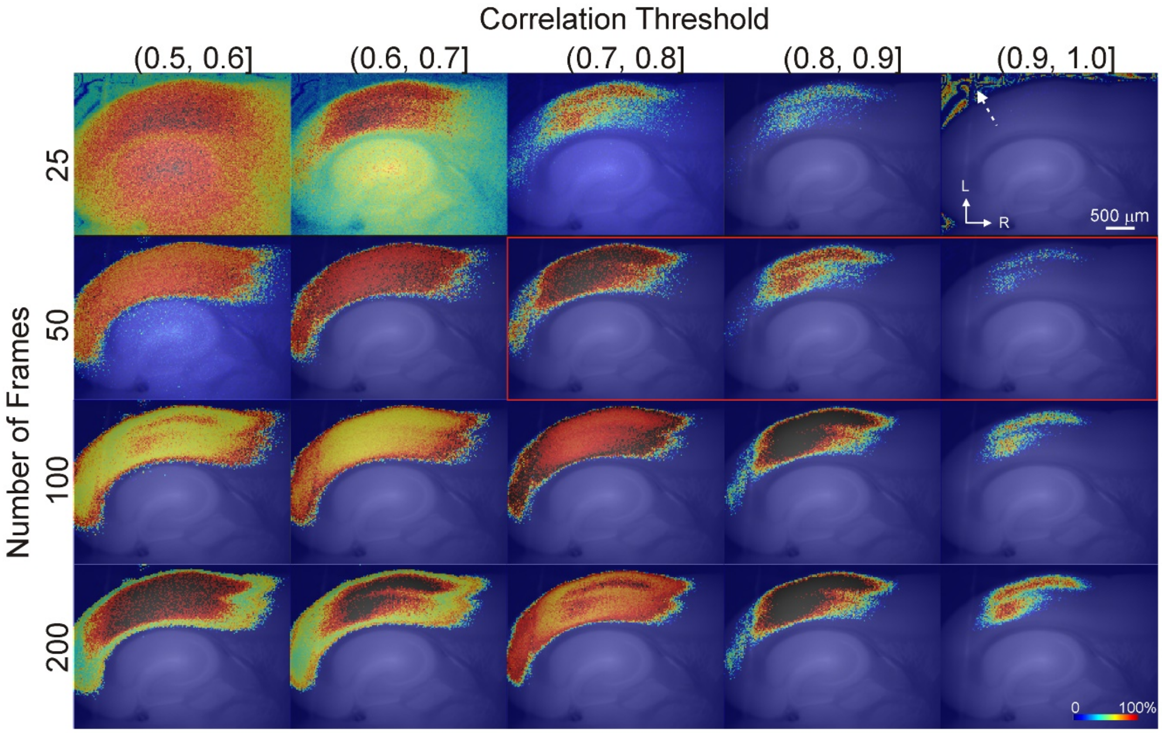

Supplemental Figure 1: Example of systematic exploration of the impact of sliding window size and correlation threshold on the detection of activated nodes during a paroxysmal activation. The impact of correlation thresholds ranging from 0.5 to 1.0 were examined in 0.1 unit increments, corresponding to the five columns shown in the image. Sliding window sizes were also adjusted from 25 to 200 frames. It was determined that the smallest window that provided adequate SNR was 50 frames with correlation threshold of greater than 0.7, highlighted by red box. Larger window sizes also produced strong SNR, but had lower temporal resolution. Shorter windows (25 frames) captured few activated nodes and picked up spurious activations outside of the slice (denoted by arrow). Images are normalized to maximum fluorescence intensity of the slice time series.

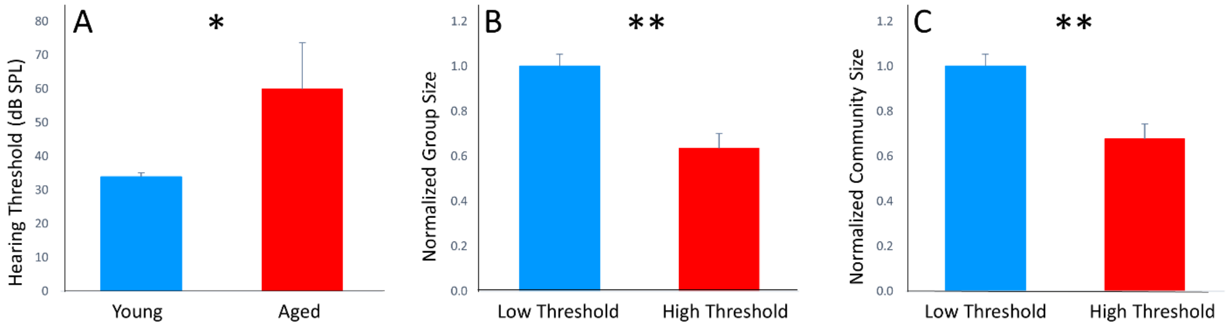

Supplemental Figure 2: A) Difference in average hearing threshold, as determined using ABR testing, for young and aged mice. B) Difference in normalized group size for activations obtained from mice with low ABR thresholds (<45 dB SPL) compared to those with high ABR thresholds ($\geq$45 dB SPL). C) Difference in normalized group size for activations obtained from mice with low ABR thresholds (<45 dB SPL) compared to those with high ABR thresholds ($\geq$45 dB SPL). *p<0.05.

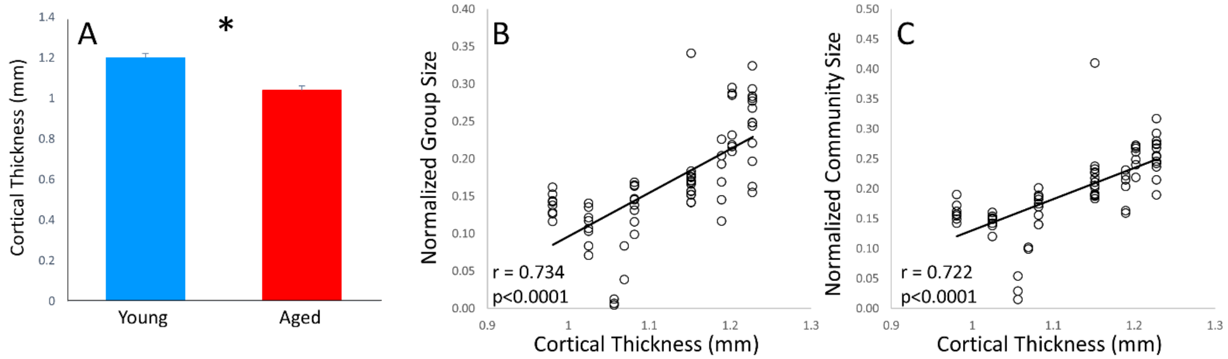

Supplemental Figure 3: A) Difference in average cortical thickness, for young and aged mice. B) Correlations between cortical thickness and normalized group size. C) Correlations between cortical thickness and normalized community size. Spearman's rho values provide for C and D. *p<0.05.

Supplemental Table 1: Output of linear mixed model multivariate regression incorporating age, cortical thickness and hearing threshold as predictors for normalized group size and normalized community size.

	Predictor	Beta	SE	t-value	p-value
**Normalized Community Sze**	Age	0.072	0.027	2.628	0.011
	Cortical thickness	0.159	0.144	1.107	0.273
	Hearing Threshold	-0.008	0.016	-0.475	0.637
**Normalized Group Size**	Age	0.057	0.032	1.793	0.078
	Cortical thickness	0.342	0.168	2.040	0.046
	Hearing Threshold	-0.018	0.019	-0.954	0.344
